# Comparison of the Fecal Microbiota in Feral and Domestic Goats

**DOI:** 10.3390/genes3010001

**Published:** 2011-12-21

**Authors:** Kassandra M. De Jesús-Laboy, Filipa Godoy-Vitorino, Yvette M. Piceno, Lauren M. Tom, Ida G. Pantoja-Feliciano, Michelle J. Rivera-Rivera, Gary L. Andersen, María G. Domínguez-Bello

**Affiliations:** 1 Microbial Ecology Laboratory, JGD 224, Department of Biology, University of Puerto Rico, Río Piedras Campus, Ave Ponce de León, San Juan, PR 00931, USA; E-Mails: kzandra.m@gmail.com (K.M.D.J.-L.); filipagodoyvitorino@gmail.com (F.G.-V.); idapantoja@gmail.com (I.G.P.-F.); yersiniamjr@hotmail.com (M.J.R.-R.); 2 Department of Ecology, Earth Sciences Division, Lawrence Berkley National Laboratory, 1 Cyclotron Rd., MS 90-1116, Berkley, CA 94720, USA; E-Mails: ympiceno@lbl.gov (Y.M.P.); ltom@lbl.gov (L.M.T.); glandersen@lbl.gov (G.L.A.)

**Keywords:** feral, domestic, microbiome, antibiotic, resistance

## Abstract

Animals have co-evolved with mutualistic microbial communities, known as the microbiota, which are essential for organ development and function. We hypothesize that modern animal husbandry practices exert an impact on the intestinal microbiota. In this study, we compared the structure of the fecal microbiota between feral and domestic goats using the G2 PhyloChip and assessed the presence of five tetracycline resistance genes [*tet*(M), *tet*(S), *tet*(O), *tet*(Q) and *tet*(W)] by PCR. Feces were collected from 10 goats: 5 domestic from a farm in the main island of Puerto Rico and 5 feral from the remote dry island of Mona. There were 42 bacterial phyla from 153 families detected in the goats’ feces. A total of 84 PhyloChip-OTUs were different in the fecal microbiota of feral and domestic goat. Both feral and domestic goats carried antibiotic resistance genes *tet*(O) and *tet*(W), but domestic goats additionally carried *tet*(Q). Diet, host genetics and antibiotic exposure are likely determinant factors in shaping the intestinal microbiota and may explain the differences observed between feral and domestic goats fecal microbiota.

## 1. Introduction

Animals have co-evolved with a microbial component that outnumbers the cells in the body of the host [1]. The microbiota is known to provide genes that contribute to important functions in the colonized organs, ranging from digestion [2], to protection against pathogens [3], development of the immune system [4], and endocrine functions [5,6].

Numerous selective forces have influenced microbial-host co-evolution shaping gut microbial diversity. Several factors may explain variations of the gut microbiota between individuals, including genotype [7], immune system [8], diet [9] and the initial colonizing microbial—founder-communities [10]. Domestication of animals during the last 10,000 years [11] has likely had an important effect in shaping the genomes of both hosts [12,13] and their microbes. Antibiotics—including tetracyclines, bacitracin, erythromycin, lincomycin, neomycin, penicillin, streptomycin, tylosin and virginiamycin—have been used in intensive agricultural systems [14] for prophylaxis and growth promotion [15]. Tetracycline is one of the most commonly used antibiotic, because of its low price and broad-spectrum activity. In 1997, the United States used a total of 2,294 tons of Tetracycline in the veterinary sector alone. 

We hypothesize that domestication has had an impact on the microbiome of animals. The Spaniards introduced goats and pigs to the Americas nearly five centuries ago [16,17], and feral animals remain in several Caribbean islands without domestication pressures. We predict that, in relation to domestic goats, feral goats have different fecal bacterial communities and fewer antibiotic resistance genes. We also expect that community distances within each group are lower than between the two groups. Artificial diets [18], herd artificial selection, and most importantly, antibiotic use [19] might have impacted the microbiota of domestic animals. To test our hypothesis, we determined the structure of the fecal microbiota and assessed the presence of some tetracycline resistance genes in feral and domestic goats.

## 2. Results and Discussion

### 2.1. Fecal Bacterial Community Structures Differ Between Domestic and Feral Goats

**Figure 1:**
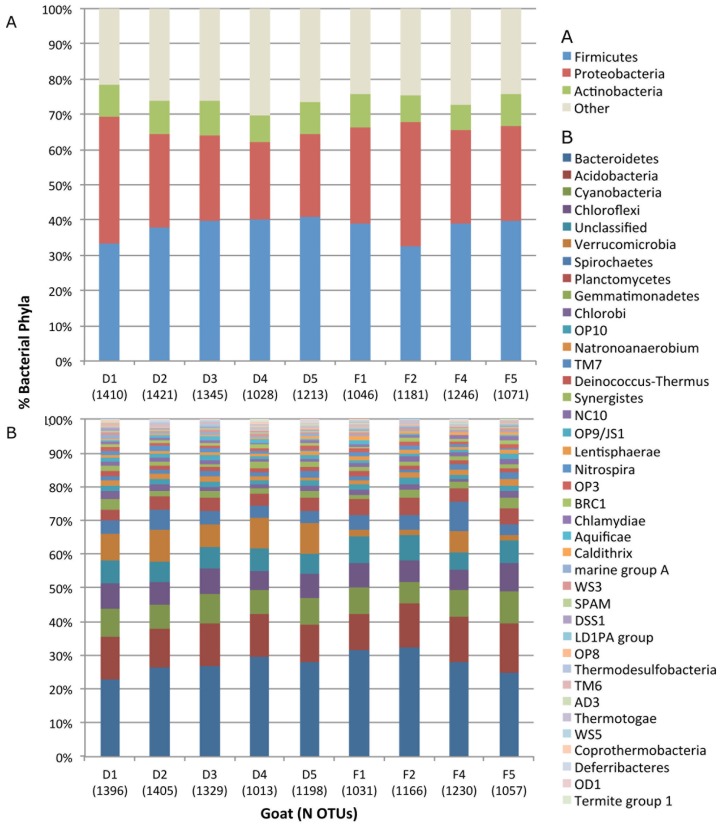
Richness distribution of the 1,982 OTUs in 42 bacterial phyla among the feces of five domestic (D) and four feral (F) goats. (**A**) Abundant phyla. (**B**) Phyla less represented (*i.e.*, ‘other’).

**Figure S1:**
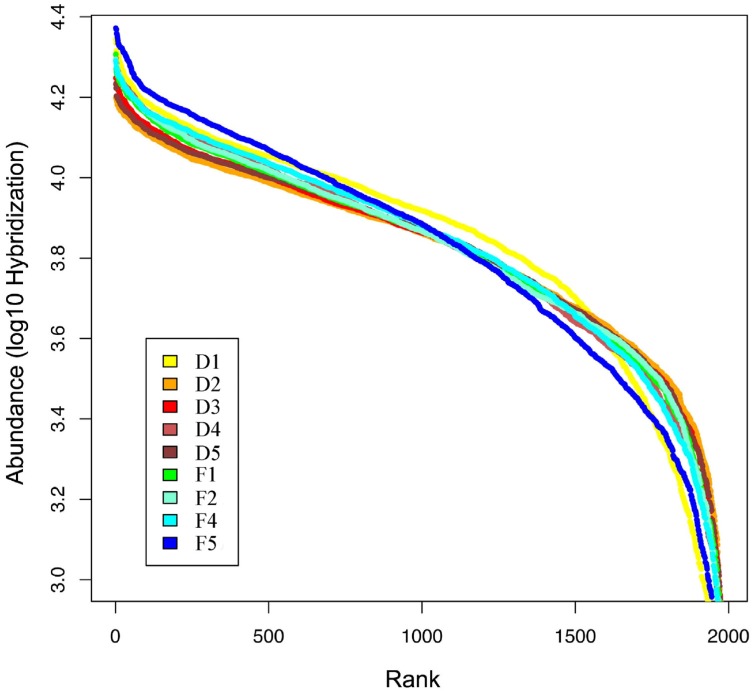
Bacterial Rank-abundance curves for fecal bacteria in domestic (D1-5) and feral (F1-5) goats.

We used a high-density 16S rDNA microarray, the PhyloChip, to study the intestinal bacterial community structure of domestic and feral goats and despite the low number of animals one (goat D1) appears to be an outlier. The PhyloChip identified bacteria belonging to 42 phyla from 153 families (Table 1 and Table S1), with many OTUs detected in the Firmicutes (35%), Proteobacteria (33%), and Actinobacteria (9%) (Figure 1). Globally, at the phylum level, the composition of the bacterial community in goat feces appeared similar among all goats, regardless lifestyle (Figure 1), and to the fecal bacterial composition in other mammals [1]. Similarities at the phylum level are consistent with those at the OTU-level, also showing no differences in bacterial rank abundance between goat groups (Figure S1).

**Table 1:** Number of bacterial taxonomic groups (OTUs ± s.e.) in feces from feral and domestic goats.

Taxonomic level	Feral goats (n = 4)	Domestic goats (n = 5)	Total N
Phylum	38 ± 1	40 ± 1	42
Class	45 ± 1	48 ± 1	52
Order	73 ± 2	76 ± 3	92
Family	157 ± 1	132 ± 4	153
Subfamily	290 ± 28	335 ± 46	548
OTUs	1,121 ± 47	1,268 ± 74	1,982

**Figure 2:**
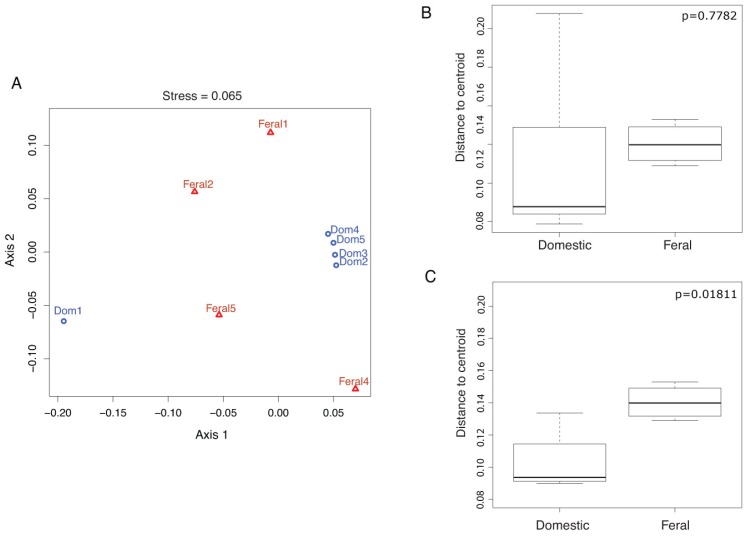
Bacterial Community Structure. (**A**) Non-metric multidimensional scaling (NMDS) of community structure in feral and domesticated goats at the PhyloChip-OTU-level; blue circles represent the domestic animals, while red triangles represent feral goats. The stress value is presented as a metaMDS stress. (**B**) Analyses of dispersion for the communities within each animal group. (**C**) Analyses of dispersion for each goat group without the domestic outlier Dom1.

**Figure S2:**
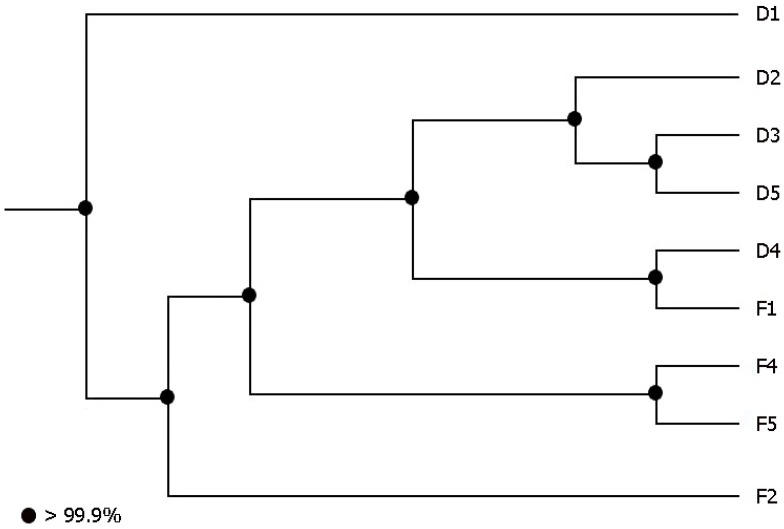
UniFrac clustering of fecal bacterial communities in domestic and feral goats. (Jackknifing node support represented by black circles) Excluding D1, an outlier, the fecal bacterial communities of domestic and feral goats largely cluster separately.

**Figure S3:**
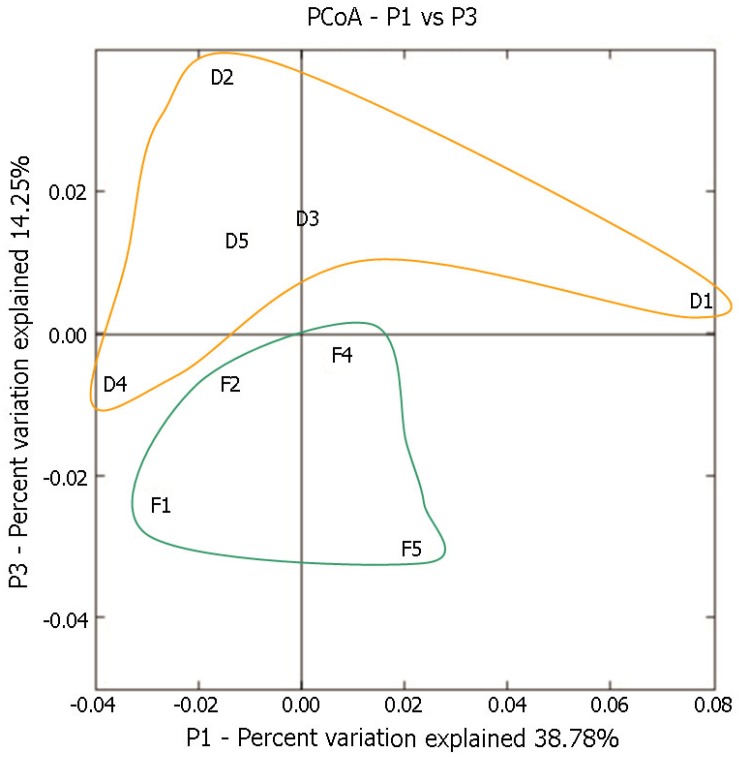
Principal coordinates analysis (PCoA): *depicting the fecal bacterial communities of five domestic (D) and four feral (F) goats using weighted UniFrac distances.* Each point corresponds to a community coded according to the goat group. The percentage of variation explained by the plotted principal coordinates is indicated on the axes. Emphasis of domestic and feral goat-associated community clustering indicated by the lines around each group.

**Figure S4:**
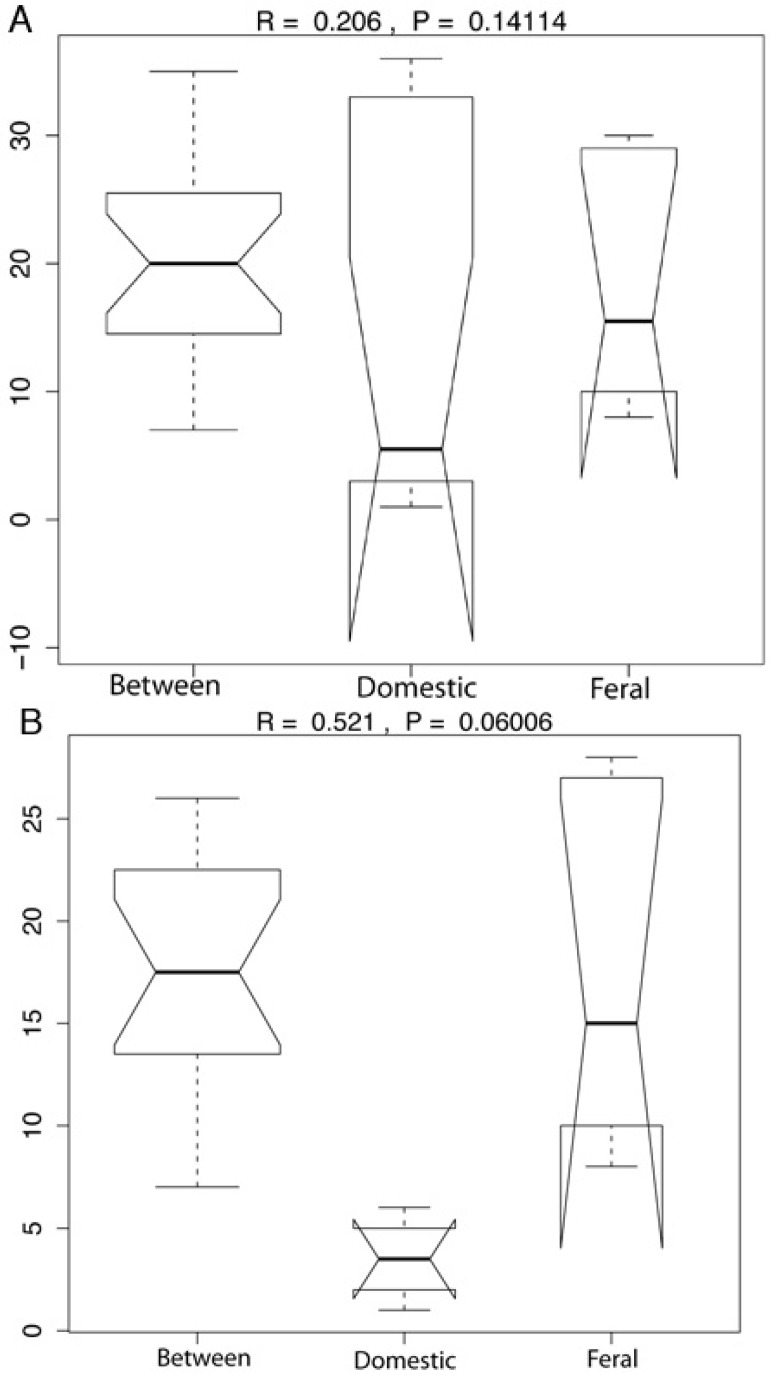
Analyses of similarities (ANOSIM) with dissimilarity ranks between and within domestic and feral goats. A) Boxplots of analyses of dissimilarity ranks. B) Boxplots of analyses of dissimilarity ranks without one domestic group outlier.

Beta diversity analyses at the PhyloChip-OTU-level using non-metric multidimensional scaling (NMDS, Figure 2(A)), UniFrac clustering (Figure S2) and PCoA (Figure S3) show that there was a clustering by goat group. However, the UniFrac significance test comparing pairwise distances showed no significant differences (p = 0.36) and the analysis of similarity using dissimilarity ranks, ANOSIM, showed only borderline significance (p = 0.141; R = 0.206), suggesting the lack of substantial dissimilarities between the fecal microbial communities of domestic and feral goats (Figure S4 (A)). Excluding D1 as an outlier (Figure S4 (B)) increases the significance of the inter-group differences (p = 0.06; R = 0.521), suggesting that inter-individual distances between goat groups are higher than those within each group. 

The results of analysis of dispersion (Figure 2(B, C)) showed higher variance in the domestic goats group (Figure 2(B)). However, when excluding the outlier domestic goat (Figure 2(C)) the group differences appear largely due to the communities of domestic goats being less variable than those of feral goats. Clostridiaceae, Bacillaceae, Lachnospiraceae and Enterobacteriaceae OTUs were the most variable families detected among feral goats that contribute to a higher variance in feral, than domestic goats.

**Figure 3:**
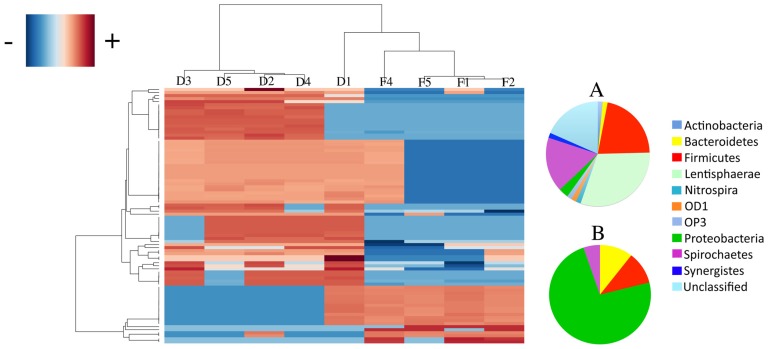
Hierarchical clustering and heatmap of the 84 OTUs that significantly differ between feral and domesticated goats. On the right, the pie charts depict phylum level distributions between bacterial taxa that were highly abundant in domestic (**A**) and in feral (**B**) goats.

To detect specific taxa responsible for suggested group differences we performed an ANOVA test (based on the quality of the means of richness and relative OTU abundance), which indicated 84 (4.2%) PhyloChip-OTUs accounting for group differences (Figure 3; Table S2). The differing OTUs belonged to 34 families and 11 phyla (Table S3). Domestic goats had higher representation of 28 of the 34 families that differed between feral and domestic goats. Bacterial families overrepresented in domestic goats belonged to Actinobacteria (7 of 7 families), Bacteroidetes (2 of 3 families), Firmicutes (4 of 4 families) and Proteobacteria (9 of 13 families), among others (Table S3). Feral goats were enriched in Proteobacteria (5 of 13 families), Bacteroidetes (1 of 3 families) and Nitrospira [(1 of 1 family); (Table S3)]. 

### 2.2. Diet and Selective Breeding Might Affect Gut Bacterial Community Structure

Environmental factors such as diet [9] and genetic factors [7] might underlie the microbiome differences in the gut communities of feral and domestic goats. Diet is an important determinant of the structure of intestinal communities [20]. Feral ungulates are browsers that consume 86 plant species in Mona island, mostly leguminous vines, canopy species or tree species from the intermediate forest stratum [21], while domestic goats, however, are fed hay and animal feedstuff. The exclusive presence of Prevotellaceae OTUs in feral goats (Table S3), which include fiber-degrading bacteria, may be related to the natural plant-based diet with a content of hemicellulose higher than the artificial feedstuff consumed by the domestic goats. The greater abundance of Bradyrhizobiaceae (nitrogen fixing soil bacteria) and Nitrospiraceae (nitrifying bacteria) OTUs in feral goats (Table S3) is consistent with the grazing of these animals (in Mona island), with possible ingestion of legume plants and soil, which may contain these bacterial groups. Other factors affecting the animal microbiome might include climate and animal ranges. Animals in Mona Island exercise freely, and are exposed directly to the harsh climate conditions of a desert island, which might lead to dehydration. In contrast, domestic animals are confined, and provided with shelter, food and water. 

In addition, geographical isolation of feral goats in Mona Island might have led to inbreeding and the consequent decrease in genetic diversity of their gut microbial communities, in relation to domestic goats. Host genetics has been altered in domestic goats, since breeders select individuals with improved production performance [12]. The evolutionary development and domestication processes determine the genetic diversity of animal species [13]. 

### 2.3. Goat’s Gut Bacteria as a Reservoir of Antibiotic Resistance Genes

In our study, all animals harbored fecal *tet*(O) and *tet*(W) genes (both in the digestive tract of swine and cows [22]; Table 2). The exclusive presence of *tet*(Q) in domestic goats is consistent with higher antibiotic exposure in farm animals. Interestingly, *tet*(M), found in human gut bifidobacteria [23], pig gut streptococci [22] and cow rumen bacteria [22], was absent in all goats in this study. They also lacked *tet*(S), a gene typical of human oral bacteria [24]. 

**Table 2:** Detection of Tetracycline resistance genes in feces from feral and domestic goats.

Goats	*tet*(M)	*tet*(S)	*tet*(O)	*tet*(Q)	*tet*(W)
Feral (n = 5)	0	0	5	0	5
Domestic (n = 5)	0	0	5	5	5

The results of this study are consistent with those in other studies comparing captive and feral [25] or wild animals [26,27], showing that domestic or urban animals have higher antibiotic resistance. The presence of antibiotic resistant bacteria in wild and feral animals [25,26,28,29] suggests that either the natural baseline of antibiotic resistance in pristine environments is not zero or that the wild environments are not completely pristine. However, antibiotics are not the only compounds that select for antibiotic resistance genes in bacteria. Bioactive compounds like Cu [30] have been found to co-select for tetracycline resistant bacteria isolated from soil.

Antibiotics can alter the intestinal environment, not only through their direct effect on bacteria, but also by affecting gut physiology. Depletion of bacteria and their microbial products that feed the colonocytes, can lead to thinning of the intestinal wall and to abnormal development of the intestine [31]. Antibiotic administration has been shown to result in a decrease in overall richness of the bacterial gastrointestinal communities in mice [19]. 

The increase in antibiotic resistance genes in the past couple of decades has been attributed to the use of antibiotics, since bacteria develop resistance when exposed to low antibiotic doses [32]. This and many other studies confirm that antibiotic resistance genes (and resistant populations) persist in the mammalian intestinal tracts even in the absence of antibiotics. The mechanisms for the persistence of these reservoirs are not clear, but it appears that the benefits for the survival of bacterial species are worth the costs.

## 3. Experimental Section

### 3.1. Animals and Samples

A total of 10 goats (*Capra hircus*) were included in the study. Five goats were from Mona Island, which is located in the Mona Passage between Hispaniola and Puerto Rico in the northeastern Caribbean (46 miles west of Puerto Rico; 18°5′12″N 67°53′22″W). It is a remote dry island with low human impact, inhabited by feral goats and pigs [21]. Five other goats were domestic goats, from a farm in Dajao, Bayamón, in the main island of Puerto Rico. Domestic animals were reported to receive Penicillin when they were sick and Daivonex as an anti-parasitic.

Fresh fecal samples were obtained from ten goats. Animals were observed to defecate and the feces were collected with a sterile spatula, carefully sampling the top part of the pellet that was not in contact with the soil. Samples were placed in microvials and immediately placed in dry ice, transported to the laboratory and stored at −70 °C for 1 month before extracting the DNA. Fecal samples were collected with permission from the Department of Natural and Environmental Resources of Puerto Rico (number 08-IC-025). 

### 3.2. DNA Extraction and Amplification

Fecal DNA was extracted using the MoBio Powersoil kit^®^, after homogenizing 250 mg feces from individual pellets in 200 µl of saline solution (0.9% NaCl), mixing in 1.5 mL tubes at high speed for 20s in a bead-beater, instead of the recommended ten minutes vortex step. The 16S rDNA was amplified using universal primers 27F (5'-AGA GTT TGA TCC TGG CTC AG-3') and 1492R (5'-GGT TAC CTT GTT ACG ACTT-3'). Each PCR mix contained 50 units/mL Taq DNA polymerase, 400 μM of each dNTP, 3 mM MgCl_2_, and 5 pmol of each primer. The PCR amplification was performed with a gradient of annealing temperatures from 48 to 58 °C. One of the feral samples failed to amplify the 16S rRNA gene. Pooled amplicons from eight different annealing temperatures were purified using the QIAquick PCR Purification Kit (Qiagen, Valencia, CA, USA) for each of the nine goats.

### 3.3. DNA Array Hybridization

We used the G2 PhyloChip 16S rDNA microarray, previously validated using qPCR and clone libraries [33,34], to characterize bacterial communities. The G2 PhyloChip microarray has 506,944 probes representing ~8,700 bacterial and archaeal taxa [33]. Although there are no sp-level taxa obtained, as with sequencing, each operational taxonomic unit (OTU) is based on an average of 25 probe pairs, each consisting of a perfectly matched and a mismatched probe, and represents 16S rDNA sequences with 0–3% sequence divergence [33]. 

The purified PCR products (200 ng) were fragmented using DNase I (0.02 Umg^−1^ DNA; Invitrogen, Carlsbad, CA, USA), biotin-labeled, and hybridized onto the PhyloChip as described by Brodie *et al* [34]. The PhyloChips were scanned using Gene Array Scanning (Affimetrix), and intensity was recorded using the standard Affymetrix software GeneChip microarray analysis suite, version 5.1. A bacterial taxon was considered present in a sample when ≥90% of the probe set designed for it was positive (positive fraction ≥ 0.9) [33]. 

### 3.4. Data and Statistical Analysis

UniFrac analyses [35] were used to compare fecal bacterial communities from feral and domestic goats, based on the phylogenetic tree, with the positive OTUs and an environment file describing the metadata for each sample, as provided by PhyloTrac [36,37]. UniFrac significance test was performed for pairwise comparisons of fecal bacterial communities using the Bonferroni correction. Jackknife environment clusters and principal coordinates analyses (PCoA) [35] were performed taking into account the relative abundances of organisms (weighted), as well as the shared branch lengths between samples. We also performed analysis of similarities (ANOSIM) using the Bray-Curtis dissimilarity, and dissimilarity ranks between and within classes were calculated and plotted. A distance matrix was calculated from the normalized log transformed intensity values of the PhyloChip-OTUs using a Bray-Curtis distance metric within the function ‘vegdist’ in the R package ‘vegan’ [38]. The distance matrix was represented as a nonmetric multidimensional scaling plot (NMDS) and the stress value (goodness of fit) was calculated, both using the function ‘metaMDS’. The relative group variance homogeneity was verified with the function ‘betadisper’ also in the “vegan” package. The boxplot function was run setting the parameter as NULL to make sure that the box was the same each time. We used additional ANOVA test to compare beta-dispersion between domestic and feral goats.

We used the statistical program “R” [39] to draw rank abundance curves from the data to visualize PhyloChip-OTU richness, overall diversity (with the “Adonis” function) and to build the heatmap with OTUs that significantly differed between feral and domestic goats as determined by ANOVA with p-values corrected for multiple observations, using the Holm procedure [40]. 

### 3.5. Detection of Tetracycline Resistance Genes

Five tetracycline ribosomal protection genes [*tet*(M), *tet*(O), *tet*(Q), *tet*(S) and *tet*(W)], were detected by PCR using specific primers [41,42] with an annealing temperature of 55 °C, except for *tet*(W) amplification which used 64 °C [43]. We used tetracycline resistance plasmids, for each of the resistance genes tested, as PCR positive controls. Each PCR mix contained 50 units/mL of Taq DNA polymerase, 400 μM of each dNTP, and 3 mM MgCl_2_. Amplicons were observed in 1% agarose gels stained with ethidium bromide (0.5 µg/mL; Bio-Rad, Hercules, CA, USA). 

## 4. Conclusions

Feral and domestic goats of Puerto Rico differed in the structure of their fecal bacterial communities, and, despite the absence of antibiotic pressures, feral goats carried fecal antibiotic resistance genes, although fewer than domestic goats. Diet, host genetic differences and antibiotic exposure might account for the differences in the microbiota between feral and domestic goats. 

## Supplementary Materials

**Table S1:** Phylum ranking, number of families and average number of bacterial OTUs (± s.e.) in domestic and feral goats.

Phylum	Phylum Ranking		N Families		Average N OTUs	
	Domestic	Feral	Domestic	Feral	Domestic (N = 5)	Feral (N = 4)
Firmicutes	1	1	25	25	485 ± 23.9	421 ± 21.9
Proteobacteria	2	2	70	59	340 ± 47.5	326 ± 31.0
Actinobacteria	3	3	31	29	115 ± 10.5	93 ± 2.1
Bacteroidetes	4	4	11	11	88 ± 4.8	82 ± 6.9
Acidobacteria	5	5	2	2	40 ± 1.7	37 ± 3.7
Spirochaetes	6	11	2	2	28 ± 2.1	9 ± 4.1
Chloroflexi	7	6	1	1	25 ± 1.4	22 ± 1.8
Unclassified	8	7	1	1	23 ± 1.3	20 ± 1.0
Verrucomicrobia	9	8	5	5	21 ± 0.6	19 ± 1.0
Cyanobacteria	10	9	1	1	14 ± 2.0	16 ± 4.6
Planctomycetes	11	10	4	4	12 ± 0.7	13 ± 0.6
Gemmatimonadetes	12	12	1	1	7 ± 0.7	6 ± 1.1
Chlorobi	13	15	2	2	6 ± 0.8	4 ± 0.7
OP10	14	14	1	1	5 ± 0.6	4 ± 0.3
TM7	15	16	1	1	5 ± 0.4	4 ± 0.9
Synergistes	16	19	1	1	5 ± 0.4	3 ± 0.3
Natronoanaerobium	17	13	1	1	4 ± 0.7	5 ± 0.5
Deinococcus-Thermus	18	17	1	1	4 ± 0.0	4 ± 0.0
OP9/JS1	19	22	1	1	4 ± 0.6	3 ± 0.5
NC10	20	18	1	1	4 ± 0.2	4 ± 0.3
Nitrospira	21	24	1	1	3 ± 0.6	3 ± 0.6
Lentisphaerae	22	20	1	1	3 ± 0.0	3 ± 0.0
OP3	23	21	1	1	3 ± 0.0	3 ± 0.0
BRC1	24	25	1	1	3 ± 0.0	3 ± 0.5
Chlamydiae	25	23	3	3	2 ± 0.2	3 ± 0.3
Aquificae	26	27	2	2	2 ± 0.4	2 ± 0.5
marine group A	27	29	1	1	2 ± 0.5	1 ± 0.3
Caldithrix	28	26	1	1	2 ± 0.0	2 ± 0.0
WS3	29	28	1	1	2 ± 0.2	2 ± 0.3
SPAM	30	30	1	1	1 ± 0.2	1 ± 0.0
DSS1	31	31	1	1	1 ± 0.2	1 ± 0.0
TM6	32	35	1	1	1 ± 0.2	1 ± 0.3
LD1PA group	33	32	1	1	1 ± 0.0	1 ± 0.0
OP8	34	33	1	1	1 ± 0.0	1 ± 0.0
Thermodesulfobacteria	35	34	1	1	1 ± 0.0	1 ± 0.0
AD3	36	36	1	1	1 ± 0.0	1 ± 0.3
Thermotogae	37	37	1	1	1 ± 0.2	1 ± 0.3
WS5	38	38	1	1	1 ± 0.2	1 ± 0.3
Coprothermobacteria	39	39	1	1	1 ± 0.2	1 ± 0.3
OD1	40	41	1	0	1 ± 0.2	0 ± 0.0
Deferribacteres	41	40	1	1	1 ± 0.2	0 ± 0.3
Termite group 1	42	42	1	0	0 ± 0.4	0 ± 0.0

**Table S2:** PhyloChip-OTUs statistical significance of ANOVA results, indicating differences between domestic and feral goats (p < 0.05).

Taxa	p-value Holm correction
Bacteria;Spirochaetes;Spirochaetes;Spirochaetales;Spirochaetaceae;sf_1;6562	3.51E-11
Bacteria;Proteobacteria;Deltaproteobacteria;Unclassified;Unclassified;sf_9;9890	8.45E-05
Bacteria;Proteobacteria;Epsilonproteobacteria;Campylobacterales;Helicobacteraceae;sf_3;10467	0.0006201
Bacteria;Actinobacteria;Actinobacteria;Actinomycetales;Thermomonosporaceae;sf_1;1546	0.00126733
Bacteria;Firmicutes;gut clone group;Unclassified;Unclassified;sf_1;4400	0.00747837
Bacteria;OP3;Unclassified;Unclassified;Unclassified;sf_2;349	0.00775145
Bacteria;Spirochaetes;Spirochaetes;Spirochaetales;Spirochaetaceae;sf_1;6506	0.00825169
Bacteria;Bacteroidetes;Bacteroidetes;Bacteroidales;Unclassified;sf_15;6233	0.00850866
Bacteria;Proteobacteria;Alphaproteobacteria;Rhodobacterales;Rhodobacteraceae;sf_1;7508	0.00853058
Bacteria;Spirochaetes;Spirochaetes;Spirochaetales;Spirochaetaceae;sf_1;6571	0.0087866
Bacteria;Spirochaetes;Spirochaetes;Spirochaetales;Spirochaetaceae;sf_1;6476	0.00905065
Bacteria;Spirochaetes;Spirochaetes;Spirochaetales;Spirochaetaceae;sf_1;6568	0.00914326
Bacteria;Spirochaetes;Spirochaetes;Spirochaetales;Spirochaetaceae;sf_1;6490	0.00938643
Bacteria;Actinobacteria;Actinobacteria;Actinomycetales;Micromonosporaceae;sf_1;1910	0.00962793
Bacteria;Actinobacteria;Actinobacteria;Actinomycetales;Micromonosporaceae;sf_1;1488	0.00962793
Bacteria;Actinobacteria;Actinobacteria;Actinomycetales;Micromonosporaceae;sf_1;1633	0.00962793
Bacteria;Actinobacteria;Actinobacteria;Actinomycetales;Micromonosporaceae;sf_1;1760	0.00962793
Bacteria;Spirochaetes;Spirochaetes;Spirochaetales;Spirochaetaceae;sf_1;6507	0.00962793
Bacteria;Synergistes;Unclassified;Unclassified;Unclassified;sf_3;117	0.00962793
Bacteria;Firmicutes;Catabacter;Unclassified;Unclassified;sf_4;4517	0.00962793
Bacteria;OD1;OP11-5;Unclassified;Unclassified;sf_1;515	0.00962793
Bacteria;Proteobacteria;Gammaproteobacteria;Alteromonadales;Alteromonadaceae;sf_1;8222	0.00962883
Bacteria;Spirochaetes;Spirochaetes;Spirochaetales;Spirochaetaceae;sf_1;6489	0.00963896
Bacteria;Actinobacteria;Actinobacteria;Actinomycetales;Kineosporiaceae;sf_1;1581	0.00966581
Bacteria;Proteobacteria;Gammaproteobacteria;Unclassified;Unclassified;sf_3;8606	0.00966755
Bacteria;Firmicutes;Clostridia;Clostridiales;Lachnospiraceae;sf_5;4324	0.00967552
Bacteria;Firmicutes;Clostridia;Clostridiales;Lachnospiraceae;sf_5;2810	0.00967554
Bacteria;Firmicutes;Clostridia;Clostridiales;Lachnospiraceae;sf_5;3223	0.00967787
Bacteria;Spirochaetes;Spirochaetes;Spirochaetales;Spirochaetaceae;sf_1;6487	0.00968213
Bacteria;Actinobacteria;Actinobacteria;Acidimicrobiales;Acidimicrobiaceae;sf_1;2014	0.00970127
Bacteria;Firmicutes;gut clone group;Unclassified;Unclassified;sf_1;4579	0.00970475
Bacteria;Actinobacteria;Actinobacteria;Actinomycetales;Gordoniaceae;sf_1;1654	0.00971853
Bacteria;Firmicutes;Clostridia;Clostridiales;Unclassified;sf_17;3099	0.00977429
Bacteria;Proteobacteria;Unclassified;Unclassified;Unclassified;sf_8;8247	0.00978004
Bacteria;Proteobacteria;Alphaproteobacteria;Bradyrhizobiales;Bradyrhizobiaceae;sf_1;7477	0.00979143
Bacteria;Proteobacteria;Betaproteobacteria;Nitrosomonadales;Nitrosomonadaceae;sf_1;7770	0.00979751
Bacteria;Proteobacteria;Deltaproteobacteria;Myxococcales;Polyangiaceae;sf_3;9671	0.00980123
Bacteria;Proteobacteria;Epsilonproteobacteria;Campylobacterales;Helicobacteraceae;sf_3;10417	0.00981157
Bacteria;Actinobacteria;Actinobacteria;Acidimicrobiales;Microthrixineae;sf_12;1721	0.00987931
Bacteria;Proteobacteria;Deltaproteobacteria;EB1021 group;Unclassified;sf_4;9741	0.00990536
Bacteria;Actinobacteria;Actinobacteria;Actinomycetales;Gordoniaceae;sf_1;1184	0.01015274
Bacteria;Proteobacteria;Gammaproteobacteria;Aeromonadales;Succinivibrionaceae;sf_1;8822	0.01020072
Bacteria;Spirochaetes;Spirochaetes;Spirochaetales;Spirochaetaceae;sf_1;6508	0.01036306
Bacteria;Spirochaetes;Spirochaetes;Spirochaetales;Spirochaetaceae;sf_1;6488	0.0104335
Bacteria;Proteobacteria;Alphaproteobacteria;Bradyrhizobiales;Bradyrhizobiaceae;sf_1;7044	0.01047121
Bacteria;Spirochaetes;Spirochaetes;Spirochaetales;Spirochaetaceae;sf_1;6523	0.01062729
Bacteria;Spirochaetes;Spirochaetes;Spirochaetales;Spirochaetaceae;sf_1;6494	0.01082364
Bacteria;Actinobacteria;Actinobacteria;Actinomycetales;Promicromonosporaceae;sf_1;1711	0.01086604
Bacteria;Proteobacteria;Alphaproteobacteria;Bradyrhizobiales;Bradyrhizobiaceae;sf_1;6799	0.01118413
Bacteria;Firmicutes;Mollicutes;Mycoplasmatales;Mycoplasmataceae;sf_1;3929	0.01120143
Bacteria;Proteobacteria;Gammaproteobacteria;Enterobacteriales;Enterobacteriaceae;sf_1;8607	0.01120143
Bacteria;Proteobacteria;Gammaproteobacteria;Thiotrichales;Thiotrichaceae;sf_3;8477	0.01120143
Bacteria;Lentisphaerae;Unclassified;Unclassified;Unclassified;sf_5;10330	0.01120143
Bacteria;Lentisphaerae;Unclassified;Unclassified;Unclassified;sf_5;9704	0.01120143
Bacteria;Unclassified;Unclassified;Unclassified;Unclassified;sf_160;2385	0.01120143
Bacteria;Firmicutes;Catabacter;Unclassified;Unclassified;sf_4;4325	0.01120143
Bacteria;Proteobacteria;Alphaproteobacteria;Rhodobacterales;Rhodobacteraceae;sf_1;7511	0.01120154
Bacteria;Nitrospira;Nitrospira;Nitrospirales;Nitrospiraceae;sf_3;833	0.01126881
Bacteria;Firmicutes;Clostridia;Clostridiales;Lachnospiraceae;sf_5;4489	0.01129829
Bacteria;Proteobacteria;Alphaproteobacteria;Rhizobiales;Bradyrhizobiaceae;sf_1;7029	0.01138694
Bacteria;Spirochaetes;Spirochaetes;Spirochaetales;Spirochaetaceae;sf_1;6479	0.01144975
Bacteria;Firmicutes;Clostridia;Clostridiales;Lachnospiraceae;sf_5;3075	0.01145728
Bacteria;Proteobacteria;Alphaproteobacteria;Bradyrhizobiales;Bradyrhizobiaceae;sf_1;6636	0.01173191
Bacteria;Proteobacteria;Alphaproteobacteria;Bradyrhizobiales;Bradyrhizobiaceae;sf_1;7522	0.01173191
Bacteria;Actinobacteria;Actinobacteria;Actinomycetales;Micromonosporaceae;sf_1;1931	0.01174823
Bacteria;Proteobacteria;Gammaproteobacteria;Enterobacteriales;Enterobacteriaceae;sf_1;8362	0.01178839
Bacteria;Bacteroidetes;Flavobacteria;Flavobacteriales;Flavobacteriaceae;sf_1;6274	0.01184388
Bacteria;Bacteroidetes;Bacteroidetes;Bacteroidales;Prevotellaceae;sf_1;5398	0.01200674
Bacteria;Proteobacteria;Alphaproteobacteria;Bradyrhizobiales;Beijerinck/Rhodoplan/Methylocyst; sf_3;7219	0.01219961
Bacteria;Proteobacteria;Alphaproteobacteria;Bradyrhizobiales;Bradyrhizobiaceae;sf_1;6768	0.01267711
Bacteria;Proteobacteria;Alphaproteobacteria;Bradyrhizobiales;Bradyrhizobiaceae;sf_1;6867	0.01287698
Bacteria;Spirochaetes;Spirochaetes;Spirochaetales;Spirochaetaceae;sf_1;6580	0.01365949
Bacteria;Firmicutes;Mollicutes;Unclassified;Unclassified;sf_1;4000	0.01386526
Bacteria;Proteobacteria;Alphaproteobacteria;Rhizobiales;Rhizobiaceae;sf_1;6770	0.0145046
Bacteria;Proteobacteria;Alphaproteobacteria;Bradyrhizobiales;Bradyrhizobiaceae;sf_1;7316	0.01458135
Bacteria;Proteobacteria;Alphaproteobacteria;Bradyrhizobiales;Bradyrhizobiaceae;sf_1;7333	0.01458135
Bacteria;Firmicutes;Clostridia;Clostridiales;Clostridiaceae;sf_12;4384	0.0149426
Bacteria;Proteobacteria;Alphaproteobacteria;Rhodobacterales;Rhodobacteraceae;sf_1;6652	0.01867578
Bacteria;Proteobacteria;Epsilonproteobacteria;Campylobacterales;Helicobacteraceae;sf_23;10443	0.02022574
Bacteria;Proteobacteria;Epsilonproteobacteria;Campylobacterales;Helicobacteraceae;sf_3;10576	0.02022574
Bacteria;Proteobacteria;Deltaproteobacteria;Myxococcales;Polyangiaceae;sf_4;9733	0.02218193
Bacteria;Proteobacteria;Alphaproteobacteria;Sphingomonadales;Sphingomonadaceae;sf_1;7215	0.0467565
Bacteria;Proteobacteria;Alphaproteobacteria;Sphingomonadales;Sphingomonadaceae;sf_1;7100	0.04689578
Bacteria;Proteobacteria;Gammaproteobacteria;Alteromonadales;Alteromonadaceae;sf_1;8978	0.04825366

**Table S3:** Taxonomic distribution of 84 OTUs overrepresented in one of the goat groups.

Phylum	Family	Domestic	Feral
Actinobacteria	Acidimicrobiaceae	**1**	0
	Gordoniaceae	**2**	0
	Kineosporiaceae	**1**	0
	Micromonosporaceae	**5**	0
	Microthrixineae	**1**	0
	Promicromonosporaceae	**1**	0
	Thermomonosporaceae	**1**	0
Bacteroidetes	Flavobacteriaceae	**1**	0
	Prevotellaceae	0	**1**
	Unclassified	**1**	0
Firmicutes	Clostridiaceae	**1**	0
	Lachnospiraceae	**4**	1
	Mycoplasmataceae	**1**	0
	Unclassified	**5**	1
Lentisphaerae	Unclassified	**2**	0
Nitrospira	Nitrospiraceae	0	**1**
OD1	Unclassified	**1**	0
OP3	Unclassified	**1**	0
Proteobacteria	Alteromonadaceae	**2**	0
	Beijerinck/Rhodoplan/Methylocyst	0	**1**
	Bradyrhizobiaceae	0	**10**
	Enterobacteriaceae	**1**	**1**
	Helicobacteraceae	**4**	0
	Nitrosomonadaceae	**1**	0
	Polyangiaceae	**2**	0
	Rhizobiaceae	0	**1**
	Rhodobacteraceae	**2**	1
	Sphingomonadaceae	**2**	0
	Succinivibrionaceae	**1**	0
	Thiotrichaceae	0	**1**
	Unclassified	**4**	0
Spirochaetes	Spirochaetaceae	**15**	0
Synergistes	Unclassified	**1**	0
Unclassified	Unclassified	**1**	0
TOTAL		**65**	19
